# Human Retinal Progenitor Cell (hRPC) Migration in Three-Dimensional (3D) Environments of Varying Stiffness and Composition

**DOI:** 10.1155/term/9963972

**Published:** 2025-10-28

**Authors:** Peng Zhao, Joydip Kundu, Douglas Blanton, Mahboobeh Rezaeeyazdi, Madeleine J. Oudin, Miles A. Miller, Aaron S. Meyer, Sidi A. Bencherif, Petr Y. Baranov, Michael J. Young, Rebecca L. Carrier

**Affiliations:** ^1^Department of Chemical Engineering, Northeastern University, Boston, Massachusetts, USA; ^2^Department of Biomedical Engineering, Tufts University, Medford, Massachusetts, USA; ^3^Department of Radiology, Harvard Medical School, Boston, Massachusetts, USA; ^4^Department of Bioengineering, University of California, Los Angeles, California, USA; ^5^Polymers, Biopolymers, Surfaces Laboratory, PBS, UMR CNRS 6270, University of Rouen Normandy, Mont-Saint-Aignan, France; ^6^Department of Ophthalmology, Harvard Medical School, Boston, Massachusetts, USA

## Abstract

Retinal degeneration is the leading cause of blindness worldwide. Subretinal implantation of human retinal progenitor cells (hRPCs) has shown great promise in models of retinal degeneration for restoration of vision but is limited by extremely low (< 2%) integration into the retina. Successful integration of implanted cells requires their migration from the site of implantation into the degenerating retina. Little is known about what cues promote RPC migration in the context of the postimplantation microenvironment, such as cues presented by a biomaterial carrier. We utilized a high-throughput assay to study the migration of hRPCs in three-dimensional hydrogel matrices of varying chemical composition and stiffness and, with exposure to different soluble factors, to identify cues important for hRPC migration and associated cell signaling events driving migration. Collagen type I, collagen type I methacrylate, and hyaluronic acid glycidyl methacrylate gels were developed with variable stiffness. The impact of key growth factors in neural development, regeneration, and cell migration such as epidermal growth factor (EGF), fibroblast growth factor (FGF), stromal cell–derived factor (SDF), and hepatocyte growth factor (HGF) was studied using hRPCs in 2 mg/mL collagen type I gels. Migration of the hRPCs varied significantly in gels of different composition and stiffness, with higher levels of mean migration distance after 48 h in nonphoto crosslinked collagen-based gels with higher concentrations of gel components and associated compressive moduli. In addition, the presence of SDF and HGF in collagen gels increased hRPC migration compared to media alone. Key signaling nodes correlating with hRPC migration were identified in Akt and MAPK signaltransduction pathways using bead-based multiplex ELISA and partial least-squares regression (PLSR) modeling. These results motivate the further exploration of material stiffness and co-delivery of soluble factors as important design parameters in cell delivery vehicles to promote transplanted hRPC migration and successful integration into degenerating retina.

## 1. Introduction

Degenerative retinal diseases like macular degeneration (MD) and retinitis pigmentosa (RP) are the leading cause of blindness in the world, with an estimated 20 million people in the United States alone suffering from loss of vision due to MD and RP [1–5]. MD is classified as “dry,” in which drusen accumulate between the choroid and retina and “wet,” in which blood vessels grow from the choroid behind the retina. MD is characterized by the death of photoreceptors in the macular region of the retina [6]. The current methods of treating MD include dietary supplements to slow progression and, for wet MD, intravitreal injections of vascular endothelial growth factor (VEGF) neutralizing monoclonal antibodies, Fc chimeric fusion proteins, or aptamers intended to reduce vascular permeability and prevent neovascularization. Photodynamic therapy, which combines drug injection and laser treatment to close abnormal blood vessels and photocoagulation, which uses laser to seal abnormal blood vessels are also employed [7, 8]. However, these treatments cannot reverse or completely stop the photoreceptor loss caused by the disease. Thus, there is an urgent need for the development of novel treatments for MD as well as other retinal degenerative diseases.

Transplantation of cells has shown promise as a treatment to replace photoreceptors lost due to MD or RP. Previous studies in animal models of retinal degeneration have shown that subretinal transplantation of retinal progenitor cells (RPCs) has the potential to result in integration of transplanted cells and restoration of vision, but the commonly applied method of bolus injection of cells in saline is associated with low cell survival and integration into the retina, likely due in part to the hostile microenvironment of the diseased retina, including nutritional support deficiency, oxidative stress, and immune rejection [9–15]. Biomaterial scaffolds with specific physical and chemical modifications have been used to deliver RPCs and have resulted in enhanced cell survival and differentiation but still low (< 10%) levels of integration [13, 16–24]. Overall, although cell transplantation-based therapy has shown promising short-term results, long-term outcomes have varied across different studies [14, 25–28]. These factors as well as additional limitations such as variability in cell source quality and surgical challenges have hindered the widespread clinical application of this method [29, 30]. Further, landmark studies within the past decade have revealed that what was previously interpreted as the integration of transplanted cells into the retina in many studies was largely protein transfer between transplanted and endogenous cells, casting uncertainty regarding the interpretation of some studies finding integration of transplanted cells [31–33]. However, multiple recent studies support the feasibility of integrating cells transplanted into the retina, as well as their formation of synapses with host retinal cells, in models of retinal degeneration [34–36]. These challenges and observations motivate consideration of what physical and chemical cues are required to promote implanted cell behaviors essential to successful integration, such as migration from the implantation site into the host retina [37]. Such cues could be incorporated within delivery vehicles for RPC transplantation.

The structure and composition of the extracellular matrix (ECM) are known to play a significant role in influencing cell behavior, and biomaterial cell carriers for transplantation essentially serve as surrogate ECM at the time of implantation. The composition of ECM-based coatings was shown to impact 2D RPC migration in a microfluidic device [37]. Matrix stiffness is an important factor impacting cell migration [1, 38, 39], and the sensitivity of cell migration to stiffness gradients may have important implications in tissue repair [40]. Here, we investigate the 3D migration of human RPCs (hRPCs) in collagen (collagen type I and collagen type I methacrylate) and hyaluronic acid (HA) gels of varying composition and stiffness. Collagen type I is mainly associated with the vasculature in the retina [41, 42], and it is one of the most abundant proteins in ECMs of various tissues throughout the body. While it is not a major component of the native retinal ECM, collagen type I is widely used in tissue engineering applications due to its ability to self-assemble into a bioactive hydrogel at physiological pH and temperature. The collagen concentration used in hydrogel formation may be varied to control pore size and scaffold stiffness, properties known to impact cell migration [1, 38, 43]. Thus, collagen type I gel can serve as a template for exploring cell responses, providing insight into how cells might behave in different ECM environments. Collagen type I methacrylate, created through the conjugation of methacrylate groups to free amines and lysine groups present on collagen type I [44], can be UV crosslinked, providing a wider range of tunable mechanical properties than collagen type I [45] and allowing for a five-fold increase in storage modulus on crosslinking [44]. The main integrins binding to the monomeric form of type I collagen are α1β1 and α2β1 integrins, while α2β1 integrin binds mainly to fibrillar collagen [46].

HA is an important component of the ECM, present in a diverse range of tissues such as skin, muscles, and cartilage, as well as retina [47, 48]. It is a major component of the interphotoreceptor matrix [49]. HA also represents a biocompatible natural polymer that can be crosslinked to produce a hydrogel for cell encapsulation, including in cell transplantation by injection to the subretinal space [47]. Expression of CD44, a major HA receptor, was observed at or around birth in some populations of mouse RPCs [50], and expression of CD44 by RPCs in vitro was demonstrated to be modulated by culture on HA [37]. Previous studies of RPC migration on ECM materials including HA demonstrated its ability to modulate cell adhesion and migration dynamics [37].

Chemical stimuli also influence cell migration, and neuronal directional migration is most frequently achieved in response to chemotactic gradients. Molecules associated with this migration include slits, netrins, and semaphorins, as well as growth factors or morphogenic proteins [1, 51]. Thus, here we also investigate the effect of four different growth factors: epidermal growth factor (EGF) [52], fibroblast growth factor (FGF) [52], hepatocyte growth factor (HGF) [53], and stromal cell–derived factor (SDF) on the migration of RPCs. These factors were selected due to their ability to promote neural cell migration [52–54]. Gradients in SDF and EGF were demonstrated to impact 2D RPC adhesion and displacement in a microfluidic system as well as migration in a Boyden chamber [55, 56]. While these studies provide important insight into factors impacting RPC migration, Boyden chambers measure migration through a filter, which is not physiologically representative, and there are known to be important differences between 2D and 3D migrations[57].

In this study, we utilized a high-throughput assay to study the 3D migration of hRPCs in hydrogel matrices of varying chemical composition and stiffness and with exposure to different growth factors. The goal was to identify cues important for hRPC migration and associated cell signaling events driving migration, as migration is one cell behavior critical in successful cell integration post transplantation. The signaling pathways governing migration were explored using bead-based multiplex ELISA for the detection of intracellular phosphoproteins significant in the MAPK and Akt pathways. This analysis was paired with partial least-squares regression (PLSR) modeling, which may offer insight into intracellular signaling events that might be triggered to better control hRPC migration.

## 2. Methods and Materials

### 2.1. hRPC Culture and Maintenance

All work with human tissue was considered as exempt from IRB approval [58]. hRPCs were isolated from human fetal neural retina at 16 weeks of gestational age at Schepens Eye Research Institute as previously described [17, 59]. The isolated hRPCs were cryofrozen and stored in a liquid nitrogen chamber. The cells were plated in tissue culture flasks (Corning, Costar) coated with fibronectin solution (100 μg/mL for 10 min, Akron Biotech) in ultraculture media (Lonza), supplemented with 2 mM L-glutamine (Invitrogen), 20 ng/mL rh bFGF (Peprotech), 10 ng/mL rh EGF (Peprotech), and 1% antibiotic-antimycotic solution  (100X, Sigma-Aldrich). This medium formulation is referred to as “RPC media” below. hRPCs were passaged at 80% confluence using Trypsin-EDTA (Gibco-Invitrogen), and cells at passage range 7–9 were used [60]. Prior to use in cell migration experiments, cells were collected using Trypsin-EDTA (Gibco-Invitrogen) and centrifuged at 400 × g for 5 min. Cells were resuspended in 1 mL media, and cell number was estimated using a hemocytometer. Cells were again centrifuged at 400 × g for 5 min and resuspended at a concentration of 4 × 10^6^ cells/mL media.

### 2.2. Cell Migration Experiments

**Figure 1:**
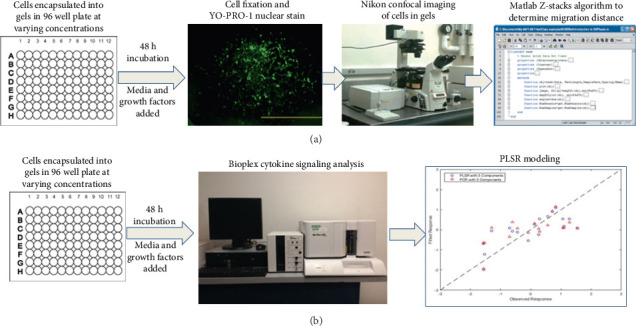
Experimental flowchart: (a) cell encapsulation in hydrogels, cell staining, and imaging and migration analysis and (b) cell encapsulation in hydrogels, Bio-Plex cell signaling assay, and PLSR modeling.

hRPC migration was studied through hydrogels of varying stiffness and composition. The study used three different hydrogel-forming materials, collagen type I, collagen type I methacrylate, and hyaluronic acid glycidyl methacrylate (HAGM), prepared as described below. In all experiments, 50 μL of hRPCs at a concentration of 4 × 10^6^ cells/mL were added to the gel precursor solution in an Eppendorf tube kept in ice and mixed via pipetting up and down to obtain the desired gel concentrations, as described below in subsequent Sections 2.2.1, 2.2.2, and 2.2.3. Immediately, 50 μL of cell-gel precursor mixture solution was added into each well of an albumin-treated 96-well tissue culture plate (Corning, Costar) kept in ice. Albumin treatment included coating 96-well plates with 50 μL of 3% bovine serum albumin (BSA, Sigma-Aldrich) overnight, removing the BSA on the day of the experiment, washing with phosphate-buffered saline (PBS, Sigma-Aldrich), and drying prior to use. After the addition of the cell-gel precursor, plates were centrifuged at 400 × g for 5 min at 4°C. After gel formation as described below for each material, 50 μL of RPC media was added on top of gels, and plates were then incubated at 37°C in cell culture incubators for 48 h as shown in Figure 1. In some wells containing collagen type I at 2 mg/mL, growth factors implicated in neuronal migration, development, and/or regeneration were added to media at concentrations previously reported to impact cell behaviors: EGF (10, 100 ng/mL), FGF (10, 100 ng/mL), SDF (10, 100 ng/mL), and HGF (10, 100 ng/mL) [61, 62]. The 2 mg/mL collagen type I gel without growth factor additions served as the negative control. It is noted that the control media also contained EGF (20 ng/mL) and bFGF (10 ng/mL), such that further addition increased the concentrations of these factors in the media. All tested groups are listed in Table 1.

#### 2.2.1. Collagen Type I

The collagen type I gels were prepared using three collagen concentrations: 8, 4, and 2 mg/mL. Prior to gel formation, collagen type I (MP Biomedical) stock solution (10 mg/mL in 20 mM acetic acid) was diluted to 8, 4, and 2 mg/mL in 10X PBS (Sigma-Aldrich), 1N NaOH (Sigma-Aldrich), and deionized (DI) water as shown in Table 2. Gels were formed by thermal crosslinking via incubation at 37°C for 30 min.

#### 2.2.2. Collagen Type I Methacrylate

Gels were prepared by diluting collagen type I methacrylate (Advanced BioMatrix, methacrylated type 1 collagen kit) stock (10 mg/mL in 20 mM acetic acid) solution to concentrations of 8, 4, and 2 mg/mL by adding to neutralization solution (alkaline 10X PBS, supplied in the kit) as per manufacturer's protocol. Uncrosslinked gels were formed by incubation at 37°C for 30 min. To prepare the photocrosslinked collagen type I methacrylate gels, the stock solution was diluted in a neutralization solution containing 20 μg/mL Irgacure 2959 photoinitiator (1:20 v/v). Gels were crosslinked by exposure to long-wave (365 nm) ultraviolet light for 15 min using a handheld crosslinker (Thermo Scientific), followed by 30 min incubation at 37°C.

#### 2.2.3. HAGM

The HA gels were prepared using two gel concentrations, 8 and 32 mg/mL. Lyophilized HAGM and acrylate-PEG-G4RGDSP (ACRL-PEG-RGD) were synthesized as previously described [63] and used to prepare the gels. Lyophilized powder of synthesized HAGM [64] was diluted in 10X PBS solution containing 20 μg/mL Irgacure 2959 photoinitiator (1:20 v/v). In some groups, to enhance cell–matrix interactions, ACRL-PEG-RGD was added to the gel precursor solutions to obtain a final concentration of 0.4% (w/v) and mixed via pipetting up and down. Gels were crosslinked by exposure to long-wave (365 nm) ultraviolet light for 15 min, followed by incubation at 37°C for 30 min.

### 2.3. Mechanical Properties

Compressive properties of hydrogels across various formulations were measured using a universal load frame (Instron Model 5542) [65]. Hydrogel samples were prepared in custom Teflon-coated molds (cylinders of 6 mm diameter by 4 mm depth) for compressive testing as previously described [66]. Uniaxial compression tests were conducted at 1 mm/min strain rate with a maximum strain at 90%. Compressive moduli were determined by the tangent of the slope of the linear region of the respective stress–strain curves. The regions between 10% and 25% strain levels of the stress–strain curves were linear across all tested hydrogel groups and selected to calculate the compressive moduli (*n* ≥ 3).

### 2.4. Imaging and Migration Analysis

After 48 h in the cell culture incubator, media was removed from the wells, and the gels were rinsed twice with 50 μL of 1X PBS. 50 μL of 4% paraformaldehyde (Sigma-Aldrich) was added to each well for 20 min, followed by additional washes with 50 μL PBS for 5 min and permeabilization with 100 μL of 0.1% Triton X-100 (Sigma-Aldrich) for 20 min. Gels were again rinsed twice with 50 μL PBS, after which 50 μL of YO-PRO-1 green nuclear dye (Molecular Probes, 1:1000 in PBS) was added for 1 h. Gels were again rinsed with 50 μL PBS for 5 min, and 10 μL PBS was left on top of the gels to keep them moist. Post-fixation, a Nikon A1R laser scanning confocal microscope with NIS software was utilized to capture 300-μm Z-stacks at 5-μm intervals using a 10× objective [67]. Stacks were captured at two different locations within each well in the 96-well plate. Imaging locations were randomly selected by confocal microscopy software and visually confirmed to be representative. Each Z-stack was then analyzed using a custom algorithm in MATLAB to detect the presence of fluorescently labeled cells in the images. The algorithm was a modified blob slice code provided by Miller [67]. The positions of the nuclei were identified relative to the well bottom (where the majority of cells reside) using nearest-neighbor analysis and principal component analysis (PCA) as previously described [67]. This algorithm determines the Z-position of the cells as the distance from the base of the plate. To note, negligible migration (5–8 μm) was observed at the beginning of the 48-h culture (time zero) across all hydrogel groups other than 8 mg/mL collagen (approximately 15 μm), confirming that hRPCs were centrifuged to the bottom of the well at time zero (Supporting Figure 1). The migration was defined as the *Z*-position difference between *t* = 48 and time zero. If the calculated difference was equal to or smaller than zero, migration was noted as not detected (nd). At least three experiments for each condition were run with over eight on-plate replicates per experiment for each of the hydrogel stiffness and growth factor stimulation experiments. To note, each data point in the figure represents the mean migration distance of a single experiment. The error bars represent the standard error of the mean. 

### 2.5. Bio-Plex Cell Signaling Assay

Samples for Bio-Plex bead-based multiplex ELISA were prepared from gels in which cell migration experiments were run as described above. At the end of the 48-h migration period, the medium above the gels was removed and replaced with 50 μL of 1 mg/mL collagenase (Advanced Biomatrix) in 1X PBS (Ca and Mg-free, Gibco) for collagen type I and collagen type I methacrylate gels and 1 mg/mL hyaluronidase (Sigma-Aldrich) in 1X PBS (Ca and Mg-free) for HAGM gels. After 1 h of digestion, the gel was triturated every 30 min for 2 h until complete dissociation. The contents of each well were pooled together for each group in 2-mL tubes and centrifuged at 1300 rpm for 10 min. The supernatant was removed and replaced with 2 mL of cell wash buffer (supplied in the Bio-Plex assay cell signaling kit). The tubes were again centrifuged, and the supernatant was removed. 150 μL of lysis buffer containing cell lysis factor QG (Bio-Rad, Bio-Plex Pro cell signaling kit) and 2 mM phenylmethylsulphonyl fluoride (PMSF) (Sigma-Aldrich) was added to each tube to obtain the cell lysate. The cell lysate was then stored at −20°C until further analysis. Protein content of the cell lysates was measured after thawing at room temperature using a micro BCA assay (Bio-Rad), and protein concentration for all samples was adjusted to 100 μg/mL using the cell lysis buffer containing 2 mM PMSF and cell lysis factor QG, as supplied by the manufacturer in the Bio-Plex assay cell signaling kit.

Bio-Plex Pro magnetic cell signaling assay was used to determine levels of phosphoproteins present in two cell signaling panels (9-plex MAPK panel, Bio-Rad, and 8-plex Akt panel, Bio-Rad), according to the manufacturer's protocol. 9-plex MAPK panel contains ATF-2 (Thr^71^), ERK1/2 (Thr^202^/Tyr^204^, Thr^185^/Tyr^187^), HSP27 (Ser^78^), JNK (Thr^183^/Tyr^185^), MEK1 (Ser^217^/Ser^221^), p38 MAPK (Thr^180^/Tyr^182^), p53 (Ser^15^), p90 RSK (Ser^380^), and STAT3 (Ser^727^). 8-plex Akt panel has Akt (Ser^473^), BAD (Ser^136^), GSK-3α/β (Ser^21^/Ser^9^), IRS-1 (Ser^636^/Ser^639^), mTOR (Ser^2248^), p70 S6 kinase (Thr^389^), PTEN (Ser^380^), and S6 ribosomal protein (Ser^235^/Ser^236^). All materials used, including lysate controls, were supplied in the kit. Briefly, 50 μL of 1X coupled magnetic beads was added to each well of the Bio-Plex 96-well flat bottom assay plate. The plates were washed twice with 200 μL of wash buffer using Bio-Rad Bio-Plex Pro II microplate wash station, and 50 μL of standards, blanks, and samples were added to the plate containing the coupled beads. Plates were covered with a sheet of sealing tape and incubated at 300 rpm for 18 h on a shaker (Orbi-Shaker, Benchmark). After the incubation period, the sealing tape was removed and the plates were washed thrice with 200 μL of wash buffer using a magnetic wash station. Subsequently, 25 μL of the detection antibodies was added to each well. The plates were covered with a new sealing tape and incubated at 300 rpm for 30 min on a shaker. After the detection antibody incubation, the plates were washed thrice with 200 μL of wash buffer using the magnetic wash station. 50 μL of streptavidin-phycoerythrin (PE) was then added to each well, and the plates were incubated at room temperature at 300 rpm for 10 min on the shaker. The plates were then washed thrice with 200 μL of wash buffer, and samples were resuspended with 125 μL of bead resuspension buffer and shaken for 30 s at 300 rpm on the shaker. The plate was then loaded into the precalibrated Bio-Plex 200 system (Bio-Rad) for analysis using the Bio-Plex Manager software version 6.0. The cell signaling assays for MAPK and Akt were performed twice in duplicate for each gel condition.

### 2.6. PLSR Modeling

The relationship between phosphoprotein measurements and measured migration distances was modeled using PLSR. PLSR can solve linear regression problems related to “cue-signal-response” relationships wherein one wishes to relate a variety of intercorrelated signals with a phenotypic outcome of interest [68, 69]. In this study, PLSR was carried out using PLS Toolbox in MATLAB software. Results from the Bio-Plex phosphoprotein signaling assay were assembled into two signaling matrices (X): *X*_1_ (10 × 18) for variable stiffness (different hydrogels) group and *X*_2_ (9 × 21) for growth factor–treated group. The columns of these matrices represent signaling metrics of 17 nodes mentioned in Section 2.5. To note, *X*_1_ had an extra column representing the compressive moduli, and *X*_2_ had four extra columns representing concentrations of the four studied growth factors (EGF, FGF, SDF, and HGF). Rows in *X*_1_ and *X*_2_ correspond to 10 hydrogel formulations described in Section 2.2 and 8 growth factor treatments (and a control with no exogenous growth factor added) with 2 mg/mL collagen type I hydrogels, respectively. The output from the cell signaling analysis contained some missing data (MD) due to sampling errors associated with low bead count. Approximately 2.3% of data points were missing, and most of the MD came from the S6 ribosomal protein measurements. MD was imputed by a trimmed score regression (TSR) algorithm developed to address MD in PLSR model building [70]. Two migration response vectors for variable stiffness (different hydrogels) group (*Y*_1_) and growth factor–treated group (*Y*_2_) were generated from the cellular migration data, with the rows corresponding to the hydrogel or growth factor conditions (10 for *Y*_1_ and 9 for *Y*_2_). The migration and phosphoprotein signaling data were mean centered and unit variance scaled prior to PLSR [71]. Two sets of modified measurements (*X*_1_‐*Y*_1_ and *X*_2_‐*Y*_2_) were then processed by MATLAB using the MathWorks plsregress function, whose outputs include loadings/scores and percentage of variance explained by the model. Loadings are the coefficients or weight vector of signaling nodes' contribution to the principal component, and scores are the magnitude of each observation along the principal component [68]. *R*^2^*Y* (coefficient of determination for the *Y* matrix) [72], *Q*^2^*Y* (fraction of the total variation in *Y* that can be predicted according to leave-one-out crossvalidation, where *k* is the row number of each input data set) [73], and variable importance in projection (VIP) [74] were calculated using output from plsregress [67, 75]. The fit and predictive capability of the model were assessed by *R*^2^*Y* and *Q*^2^*Y*, respectively, and *Q*^2^*Y* was used to determine the number of principal components [69]. Briefly, from the second principal component, with each new principal component, if the *Q*^2^*Y* increased more than 0.05, the principal component was kept and another principal component was added. If the *Q*^2^*Y* only had minimal change or decreased, addition of principal components stopped at the previous principal component [69]. VIP assessed the contribution of each variable (signaling nodes) across the entire model and indicated important variables (VIP > 1) in explaining the response vector [69]. Furthermore, a reduced PLSR model with optimally selected variables/conditions based on VIP and migration data was built to improve accuracy of migration distance predictions, as described below.

### 2.7. Statistical Analysis

GraphPad Prism (GraphPad Software Inc., San Diego, CA, version 10.0) was used to perform statistical analysis. Data were presented as mean ± standard deviation (SD). Sample sizes are reported for each experiment and differed among some groups due to technical errors and contaminations. The results of compressive modulus and migration tests were analyzed using one-way ANOVA to determine the differences between groups, where *p* < 0.05 was considered statistically significant (^∗^*p* < 0.05, ^∗∗^*p* < 0.01, ^∗∗∗^*p* < 0.001, ^∗∗∗∗^*p* < 0.0001). Prior to selecting the appropriate ANOVA model, homogeneity of variances was assessed using the Brown–Forsythe test. If the test indicated equal variances, ordinary one-wayANOVA was applied. If unequal variances were detected, Welch's one-way ANOVA was used.

## 3. Results and Discussion

### 3.1. Mechanical Properties

**Figure 2:**
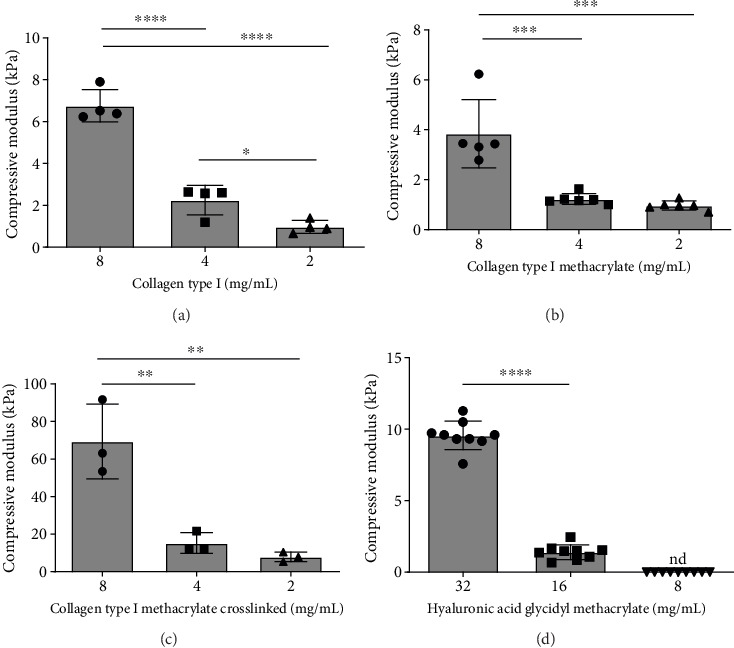
Compressive modulus measured for (a) collagen type I hydrogel, (b) collagen type I methacrylate hydrogel, (c) collagen type I methacrylate crosslinked hydrogel, and (d) HAGM hydrogel. The error bars represent the standard deviation. Statistical analysis was performed using Welch's one-way ANOVA, and the means of the different groups were compared using Dunnett's post hoc test (^∗^*p* < 0.05, ^∗∗^*p* < 0.01, ^∗∗∗^*p* < 0.001, ^∗∗∗∗^*p* < 0.0001, *n* ≥ 3).

The mechanical properties of the hydrogels studied were characterized for insight into their potential impact on RPC migration. As expected, the compressive modulus of each gel type increased with increasing concentration of the gel-forming component, as well as with crosslinking (Figure 2). The magnitude and trend of increasing compressive modulus with increasing concentration (from 2 to 8 mg/mL) were similar for collagen type I and uncrosslinked collagen type I methacrylate gels, with values ranging from approximately 1–7 kPa. To note, the compressive modulus of the 2 mg/mL collagen type I gel was close to the lower limit of the reported retinal tissue stiffness range (1–20 kPa) [76, 77]. The compressive modulus of HAGM gels increased from 1.4 to 9.6 kPa as the concentration of the gels increased from 16 to 32 mg/mL. The compressive modulus of the 8 mg/mL HAGM gels could not be determined, as the gels were found to be very soft. When methacrylated groups were crosslinked using UV irradiation to provide gels of greater stiffness, compressive modulus markedly increased relative to non-crosslinked gel groups (e.g., 43.4 and 3.84 kPa for crosslinked and non-crosslinked 8 mg/mL collagen type I methacrylate gels, respectively). The native porcine retina, which is primarily composed of neurons and their axons, has a modulus of 10.5 ± 2.67 kPa [78], comparable to the moduli of the developed collagen type I (8 mg/mL) and HAGM (32 mg/mL) gels.

### 3.2. hRPC Migration in Collagen and Composite Hydrogels With Varying Stiffness

**Figure 3:**
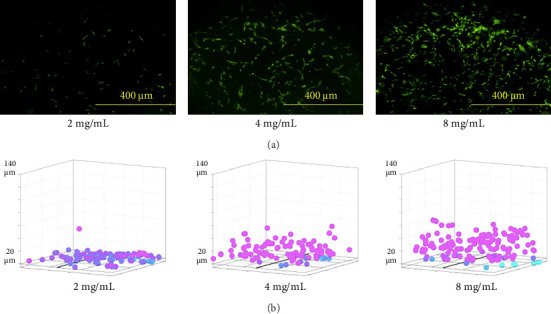
Images of RPCs after 48 h in gels. (a) Representative images of RPCs fluorescently labeled with YoPro nuclear dye within various collagen type I gels at 50-μm distance from plate bottom. (b) Scatterplots showing identified cells and measured migration within collagen type I gels with varying concentration and stiffness.

**Figure 4:**
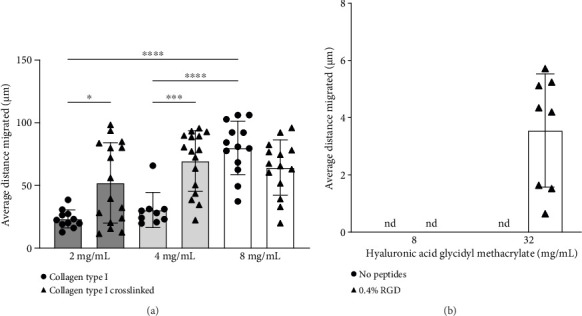
Average distance migrated measured for cells encapsulated in (a) collagen type I hydrogels and collagen type I methacrylate crosslinked hydrogels and (b) HAGM hydrogels after 48 h in culture. The error bars represent the standard deviation. Statistical analysis was performed using Welch's one-way ANOVA, and the means of the different groups were compared using Dunnett's post hoc test (^∗^*p* < 0.05, ^∗∗^*p* < 0.01, ^∗∗∗^*p* < 0.001, ^∗∗∗∗^*p* < 0.0001, *n* ≥ 3).

After 48 h, hRPCs exhibit varying degrees of migration in different hydrogel compositions (Figure 3), with a general trend of migration distance increasing with increasing compressive modulus (Figure 4, Supporting Figure 2). The average migration distance of hRPCs increased 3-fold in collagen type I gels upon increasing the gel concentration from 2 to 8 mg/mL (Figure 4(a)). In addition, there was a 2-fold increase in migration distance in crosslinked relative to non-crosslinked 2 mg/mL gels. There was no significant difference in migration distance between the different crosslinked collagen type I methacrylate concentration groups (Figure 4(a)), however, indicating there may be a limit to the potential impact of mechanical stiffness on migration. It is important to note that increasing the concentration of the gel-forming component changes not only the gel stiffness but also the density of attachment ligands and effective porosity, complicating any conclusions regarding the impact of hydrogel compressive modulus on cell migratory ability. It is possible a decrease in effective gel pore size with crosslinking inhibits migration. While, as noted, collagen type I is not a major component of the native retinal ECM [41, 42], the migration of RPCs through the collagen matrix indicates interaction with type I collagen. Migration was only observed in HAGM with incorporated RGD (Figure 4(b)).

### 3.3. hRPC Migration Induced by Chemokine Presentation

**Figure 5:**
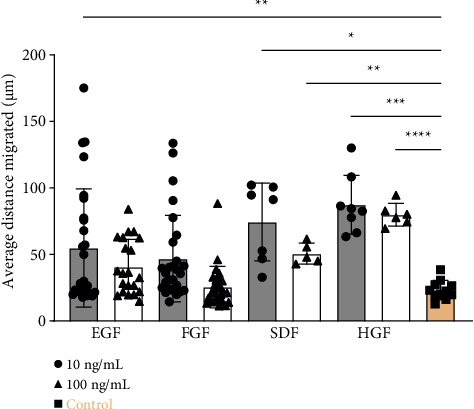
Average distance migrated by hRPCs encapsulated within 2 mg/mL collagen type I gels upon exposure to select soluble factors EGF (10, 100 ng/mL), FGF (10, 100 ng/mL), SDF (10, 100 ng/mL), and HGF (10, 100 ng/mL). The error bars represent the standard deviation. Statistical analysis was performed using Welch's one-way ANOVA, and the means of the different groups were compared to the control group (2 mg/mL collagen type I) using Dunnett's post hoc test (^∗^*p* < 0.05, ^∗∗^*p* < 0.01, ^∗∗∗^*p* < 0.001, ^∗∗∗∗^*p* < 0.0001, *n* ≥ 5).

hRPC migration in the 2 mg/mL collagen type I hydrogel was significantly enhanced by chemokines implicated in neuronal migration, regeneration, and development [51, 61, 62]. SDF and HGF (10 and 100 ng/mL) resulted in significant increases in migration distance (Figure 5), while the addition of EGF or FGF did not significantly increase the hRPC migration distance relative to the media control. These data suggest that growth factors can be used to significantly increase hRPC migration. It is noted that the growth factors tested were added to the media on top of the gel systems, rather than within the gels themselves. This could have resulted in a gradient in growth factor concentration, at least for some period of time during the migration experiment. In the context of retinal transplantation, it would be technically challenging to introduce a gradient by exposing a single side of an injected cell-laden gel to exogenous growth factors. Upon transplantation into the subretinal space, an injected hydrogel could be inherently exposed to gradients in factors secreted differentially by the retinal and subretinal (i.e., retinal pigmented epithelium) tissues. Alternatively, gradients could potentially be created by injection of soluble factors into the vitreous.

### 3.4. Phosphoprotein Analysis

**Figure 6:**
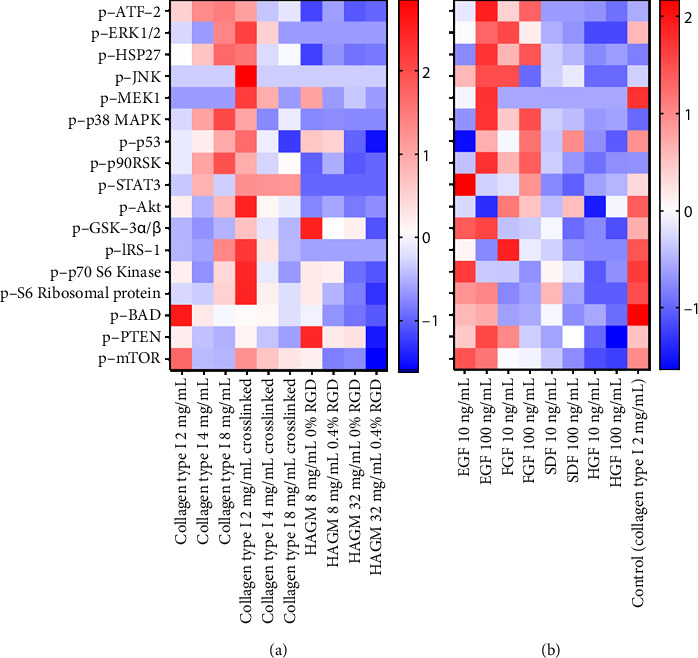
Heat maps for Bio-Plex data representing the values of each phosphoprotein present in two cell-signaling panels (9-plex MAPK panel, Bio-Rad, and 8-plex Akt panel, Bio-Rad) analyzed for each gel condition in which cell migration experiments were run. Data were mean centered and unit variance scaled. Color bar indicates intensity levels for mean-centered and unit variance–scaled data. (a) Heat map of phosphoprotein data in groups with varying hydrogel stiffness/composition. (b) Heat map of phosphoprotein data in growth factor–treated groups.

Bead-based multiplex ELISA was used to measure the concentration of 17 proteins known to be involved in MAPK and Akt signaling pathways in hRPCs after they were cultured for 48 h within the gels. The MAPK and Akt pathways have been extensively explored as central regulators of cell behaviors [79, 80]. The MAPK signaling pathway is significant in migration of multiple cell types, including RPC stimulated with EGF [55, 81]. Akt signaling has also been reported to generally play significant roles in migratory processes of multiple cell types [82]. Furthermore, interplay between MAPK and Akt pathways makes them a powerful pair to study together [83, 84]. Heat maps for Bio-Plex data representing phosphoprotein values present in two cell-signaling panels (9-plex MAPK panel and 8-plex Akt panel) analyzed for each gel condition are presented in Figure 6. The heat map analysis suggests that varying the scaffold stiffness and composition (Figure 6(a)) influenced the relative mean fluorescence intensities of the 9 MAPK and 8 Akt phosphoproteins. Overall, levels of most measured phosphoproteins were higher within collagen gels than within HAGM gels, indicating more significant activation of the associated signaling pathways. For example, the average fluorescence level of p38 MAPK within collagen type I gels is approximately 12 times higher than the level within HAGM gels. Similarly, varying growth factor treatment impacted measured levels of the selected 17 nodes (Figure 6(b)). The measured levels of most studied nodes were higher in the EGF- and FGF-treated groups relative to the cells within 2 mg/mL collagen exposed to standard cell culture media, while they were lower in the SDF and HGF groups.

### 3.5. Migration-Associated Phosphoproteins and PLSR Modeling

Examination of the intensities of selected nodes in conjunction with the averaged migration data suggests that MAPK/Akt signaling pathways affected hRPC migration differently within the variable hydrogel stiffness and growth factor-treated groups. Overall, intensities of many signaling nodes were greater in hydrogels resulting in relatively long migration distances (i.e., collagen type I-based gels) relative to those with shorter migration distances (i.e., HAGM gels) (Figure 6, Supporting Figure 3). Exceptions included PTEN, which is an inhibitor of Akt signaling [85], and GSK, which is inhibited by Akt [86]. However, in the growth factor–treated groups, phosphoprotein intensities were generally lower in the groups with relatively longer migration distances (SDF and HGF) than in groups with relatively shorter migration distances (EGF and FGF) (Supporting Figure 4). In examining the phosphoprotein data, certain trends can be observed with respect to relationships between measured levels and migration. For example, intensities of specific nodes, such as ATF, HSP27, and p90RSK, were found to positively correlate with average migration distance within some hydrogel groups (including the collagen type I groups and comparing 32 mg/mL HAGM groups with and without RGD), while others were found to negatively correlate with average migration distance (BAD, PTEN, and mTOR) (Supporting Figure 3), indicating a possible role in promoting hRPC migration in these groups. However, as cell migration response is dictated by interactions among multiple signaling nodes and pathways [68], it is challenging to state which signaling nodes contribute most significantly to hRPC migration via visual observation of phosphoprotein levels across the various hydrogel conditions.

**Figure 7:**
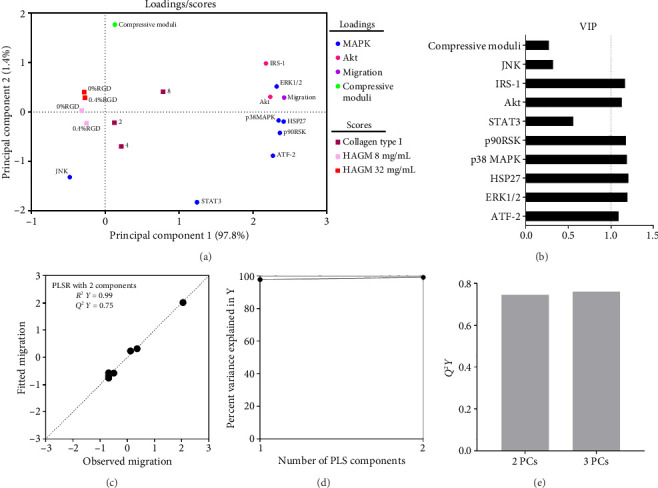
Reduced PLSR modeling with 2 principal components of relationship between select signaling nodes and cell migration in different hydrogels. (a) Projection of loadings and scores onto the first two principal components. Loadings of individual phosphoprotein nodes were plotted in blue (MAPK) and red (Akt) circles. Loading of cell migration was plotted as a purple circle. Loading of compressive moduli was plotted as a green circle. Scores of each hydrogel formulation were plotted in squares. (b) VIP scores of selected signaling nodes. (c) Predicted migration vs. observed migration. Model quality and crossvalidation prediction accuracy were determined by *R*^2^*Y* (0.99) and *Q*^2^*Y* (0.75). (d) Percent variance explained in *Y*. (e) *Q*^2^*Y* for each principal component.

PLSR modeling was used to better understand potential relationships between hRPC migration within hydrogels and MAPK/Akt signaling pathways. First, a PLSR model was built using 2 principal components (based on *Q*^2^*Y* as described in Section 2.6), where the first principal component accounts for 64.2% of the total variance and the second captured 18.1%. The model was built to describe the relationship between the signaling nodes (Figure 6(a)) and compressive moduli as inputs and migration distance across different hydrogel matrices as the output. Clustering of certain signaling nodes (i.e. ATF, p38, HSP27, p90RSK, and STAT3) and compressive moduli at the top-right quadrant of the loadings plot indicated their overall positive correlations with hRPC migration (Supporting Figure 5). These five signaling nodes exhibited larger absolute values for loading in the first principal component, indicating their stronger contributions to it. In contrast, the compressive moduli had a larger loading value in the second principal component. The VIP score (Supporting Figure 5B) indicated these nodes, as well as ERK, JNK, Akt, and IRS-1, were important for predicting hRPC migration. Many of these nodes have been reported to be associated with migration of various cell types [10]. For example, IRS-1 is reported to be closely related to epithelial cell migration [68]. ERK and HSP27 were associated with migration in both mesenchymal and epithelial cells [68]. p38 was suggested to be involved in the migration of muscle cells, porcine aortic endothelial cells, and corneal epithelial cells [10]. Recent reports indicated that p90RSK affected edge dynamics during cell migration [87]. STAT3 was found to modulate actin cytoskeleton organization and cell migration via regulating Rac1 [88]. In contrast, signaling nodes at the bottom-left region of the loadings plot (i.e., PTEN, and GSK) were negatively correlated with hRPC migration. However, GSK is known to promote cell migration in other cell types [89]. The scores of each observation were in accord with the migration results, such that hydrogels in which cell migrated over relatively longer distances (collagen type I and collagen type I crosslinked) were positive in the first and/or second principal components. While the PLSR model fitted the data well (*R*^2^*Y *= 0.82), it had limited predictive capability (*Q*^2^*Y *= 0.29), indicating overfitting (Supporting Figure 5C, 5E). Leaving uninformative variables out or increasing the training dataset could improve the predictive capability [67, 69]. Thus, a reduced PLSR model with those variables having a VIP score greater than 1 was established to provide better migration prediction accuracy using less variables (Figure 7). The 8 nodes noted above were used to build the model, and the collagen type I crosslinked groups were excluded from the training set. This exclusion was based on the observation of lack of minimal change in migration distance across these groups in spite of significant changes in compressive moduli as well as observation that many of 17 MAPK/Akt signaling node intensities in these groups were not correlated with observed hRPC migration (Supporting Figure 5). The first principal component in this reduced PLSR model captured a greater portion of the total variance (97.8%) relative to the original model. The loading values of nearly all the selected nodes (except for JNK) in the first principal component remained positive. However, the loadings of some nodes in the second principal component shifted from positive to negative. Previously clustered nodes, including p38, p90RSK, HSP27, and ATF, remained together in the reduced model, but their positions shifted from the top-right quadrant to the bottom-right quadrant. The predictive capability as well as the quality were improved (*R*^2^*Y *= 0.99, *Q*^2^*Y *= 0.75) in the reduced PLSR model (Figures 7(c), 7(e)).

**Figure 8:**
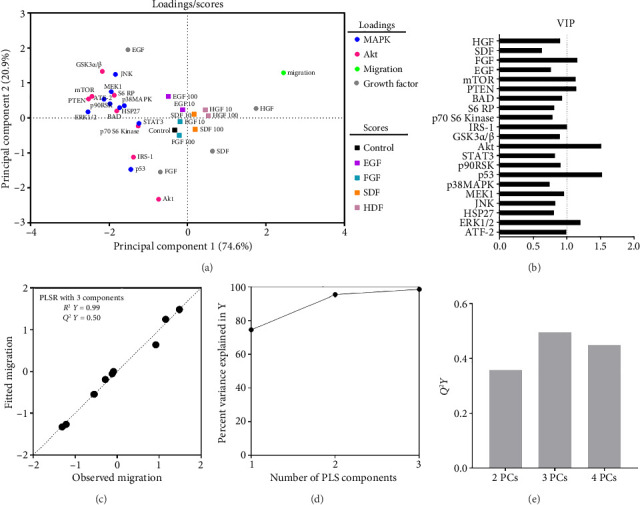
PLSR modeling with 3 principal components of growth factor–treated groups. (a) Projection of loadings and scores onto the first two principal components. Loadings of individual phosphoprotein nodes were plotted in blue (MAPK) and red (Akt) circles. Loading of cell migration was plotted as a green circle. Scores of each growth factor were plotted in squares. (b) VIP scores of 17 signaling nodes and compressive moduli. (c) Predicted migration vs. observed migration. Model quality and crossvalidation prediction accuracy were determined by *R*^2^*Y* (0.99) and *Q*^2^*Y* (0.50). (d) Percent variance explained in *Y*. (e) *Q*^2^*Y* for each principal component.

Next, a PLSR model was built using 3 principal components to describe relationships between MAPK/Akt signaling (Figure 6(b)) and migration response of hRPC under various growth factor treatments (Figure 8). The first and second principal components accounted for 74.6% and 20.9% of the variance, respectively. Almost all signaling nodes were found to be located at the left side of the loadings plot (negative in the first principal component) and 5 nodes located at the lower-left quadrant (Figure 8(a)), indicating that most signaling nodes correlated negatively with hRPC migration under the growth factor treatments investigated. These loading results were in accordance with observed higher signaling node measurements in many groups with shorter migration distance (Figure 6(b), Supporting Figure 4). Many of the lower-left region nodes have been reported to positively impact the migration of other cell types, supporting the importance of context in interpreting cell response to stimuli [68, 89]. For growth factor nodes, only SDF and HGF were positive in the first principal component, consistent with our observation that these growth factors most potently induced migration, as noted in Section 3.3. These findings are in agreement with previous reports indicating the significance of SDF in 2D RPC migration and the role of HGF in migration of other neuronal cells [53, 90]. The quality and predictive capability of the developed model were good (*R*^2^*Y *= 0.99 and *Q*^2^*Y *= 0.50) (Figures 8(c), 8(e)).

One possible way to further improve the model's hRPC migration prediction is to include basal phosphoprotein intensities prior to the growth factor stimulus (initial time points) to consider growth factor–independent effects (background) on the hRPC migration [68]. Also, looking at the dynamics of signaling responses of selected nodes can help provide deeper insights into intracellular signaling events of hRPC migration [68].

### 3.6. Limitations and Future Directions

While fetal-derived RPCs were used in these studies, it is noted that induced pluripotent stem cell (iPSC)–derived retinal cells have been used in multiple clinical trials involving cell transplantation[14]. The migration behaviors of iPSC-derived retinal cells might differ considerably from those of fetal-derived retinal cells. Further, examining cell migration in hydrogels that more closely resemble the retinal ECM composition and over longer time scales relevant to response to cell transplantation could provide significant insight. It should also be noted that* in vivo *conditions, including ECM components as well as tissue architecture and interactions with other cell types are likely to significantly influence the extent of cell migration in response to materials and growth factors. Furthermore, exploring other signaling pathways, such as CXCR4 and Wnt, may enhance understanding of the mechanisms driving hRPC migration. Other cell responses including apoptosis, proliferation, and differentiation, which are highly relevant to success of cell transplantation therapies and could also be affected by biomaterial cues and regulated by pathways that involve or intersect with the MAPK and Akt pathways, should be explored in the future. Finally, exploring cell migration in a more physiologically relevant model (e.g., an explant model) could offer a deeper understanding of the migration behaviors of implanted cells post-transplantation.

## 4. Conclusion

In this study, we explored the migration potential of hRPCs in 3D hydrogel matrices, providing insight into physicochemical cues that may be explored to enhance the integration of retinal precursors within the host retina following subretinal transplantation and thus transplantation success. Herein, we quantified the differences in migration for hRPCs when seeded in gels of varying composition with associated varying stiffness, as well as with varying concentrations of growth factors. Our results indicate gel mechanical properties and exposure to growth factors impact the migration of hRPCs. Specifically, stiffer gels (i.e., compressive modulus of approximately 7 kPa vs. 1 kPa) as well as the presence ofSDF and HGF in the range of 10–100 ng/mL enhance migration. Using multiplex ELISA and PLSR modeling, we have determined signaling nodes correlated with migration distance. Overall, this work demonstrates a strategy for quantifying RPC migration potential, which could be more directly compared to transplant effectiveness in future studies. It also motivates consideration of the mechanical properties of cell delivery vehicles and further exploration of specific signaling cascades that may be targeted by soluble factors to maximize hRPC migration upon implantation and ultimately facilitate retinal tissue repair.

## Figures and Tables

**Table 1:** Tested groups.

Material	Concentration (mg/mL)	RGD
Collagen type I	2	0
Collagen type I	4	0
Collagen type I	8	0
Collagen type I methacrylate	2	0
Collagen type I methacrylate	4	0
Collagen type I methacrylate	8	0
HAGM	8	0
HAGM	32	0.4%
HAGM	8	0
HAGM	32	0.4%

**Material**	**Growth factor**	**Concentration (ng/mL)**

Collagen type I	EGF	10
Collagen type I	EGF	100
Collagen type I	FGF	10
Collagen type I	FGF	100
Collagen type I	SDF	10
Collagen type I	SDF	100
Collagen type I	HGF	10
Collagen type I	HGF	100

*Note:* Each row represents a hydrogel formulation.

**Table 2:** Formulation table for preparing 1 mL of collagen type I gel precursor solution.

	Collagen gel concentration		
	8 mg/mL	4 mg/mL	2 mg/mL
Collagen type I^∗^	800 µL	400 µL	200 µL
10*X* PBS	100 µL	100 µL	100 µL
1N NaOH	40 µL	20 µL	10 µL
DI water	10 µL	430 µL	640 µL
Cell suspension^∗∗^	50 µL	50 µL	50 µL
Total volume	1000 µL	1000 µL	1000 µL

^∗^Stock concentration of collagen type I is 10 mg/mL.

^∗∗^Cell density is 4 × 10^6^ cells/mL.

## Data Availability

The datasets generated and/or analyzed during the current study are available within this published article. All data and materials are available from the corresponding author on reasonable request.

## Supporting Information

The following supporting information is available to support the findings of this study:

This file contains Supporting Figures 1–5 mentioned in the main text. Supporting Figure 1 showed time zero hRPC location and calculated hRPC migration within all hydrogels. Supporting Figure 2 displays representative images of YO-PRO-1-labeled hRPCs within collagen type I hydrogels. Supporting Figure 3 shows Bio-Plex data of 17 signaling nodes plotted with averaged migration distance in different hydrogels (collagen type I, collagen type I crosslinked, and HAGM). Supporting Figure 4 presents Bio-Plex data of 17 signaling nodes plotted with averaged migration distance in 2 mg/mL collagen type I hydrogels treated with different growth factors (EGF, FGF, SDF, and HGF). Supporting Figure 5 displays PLSR modeling with 2 principal components of impact of signaling nodes on migration distance in different hydrogel matrices (collagen type I, collagen type I crosslinked, and HAGM).
