# Supplementary material for: Human Retinal Progenitor Cell (hRPC) Migration in Three-Dimensional (3D) Environments of Varying Stiffness and Composition
Source: J Tissue Eng Regen Med. 2025 Oct 28;2025:9963972. doi: 10.1155/term/9963972 (PMC12585844; doi:10.1155/term/9963972)
Supplement: Supporting Information — Additional supporting information can be found online in the Supporting Information section. [file 9963972.f1.zip › Supplementary Material File.docx]

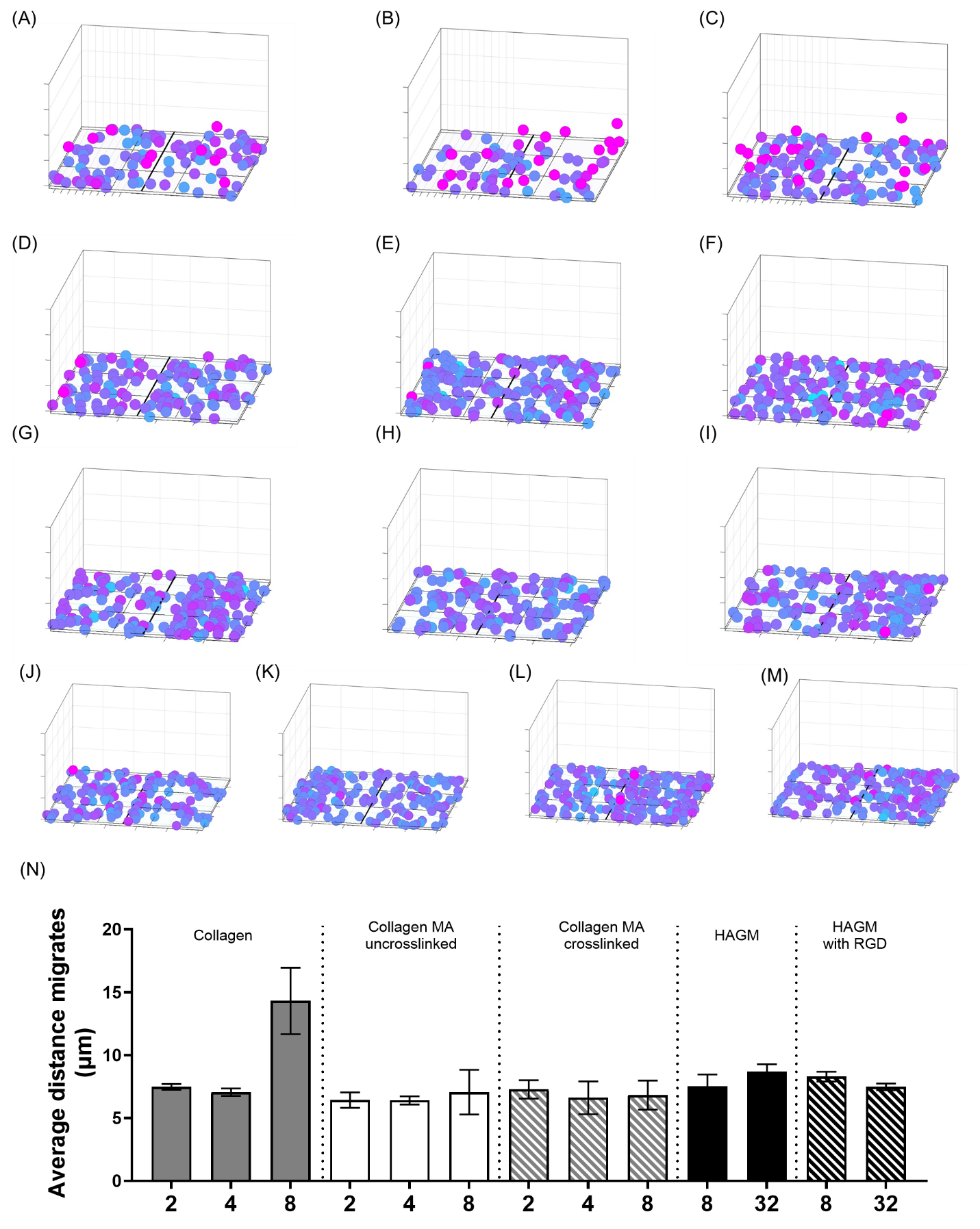


**Supplemental** **Fig. 1**. Time zero scatterplots of identified hRPCs within (**A**) 2mg/ml, (**B**) 4mg/ml, and (**C**) 8mg/ml collagen type I gels; (**D**) 2mg/ml, (**E**) 4mg/ml, and (**F**) 8mg/ml collagen type I methacrylate gels; (**G**) 2mg/ml, (**H**) 4mg/ml, and (**I**) 8mg/ml collagen type I methacrylate crosslinked gels; (**J**) 8mg/ml and (**K**) 32mg/ml hyaluronic acid glycidyl methacrylate gels, (**L**) 8mg/ml and (**M**) 32mg/ml hyaluronic acid glycidyl methacrylate gels with RGD. (**N**) Measured migration corresponding to time zero scatterplots.


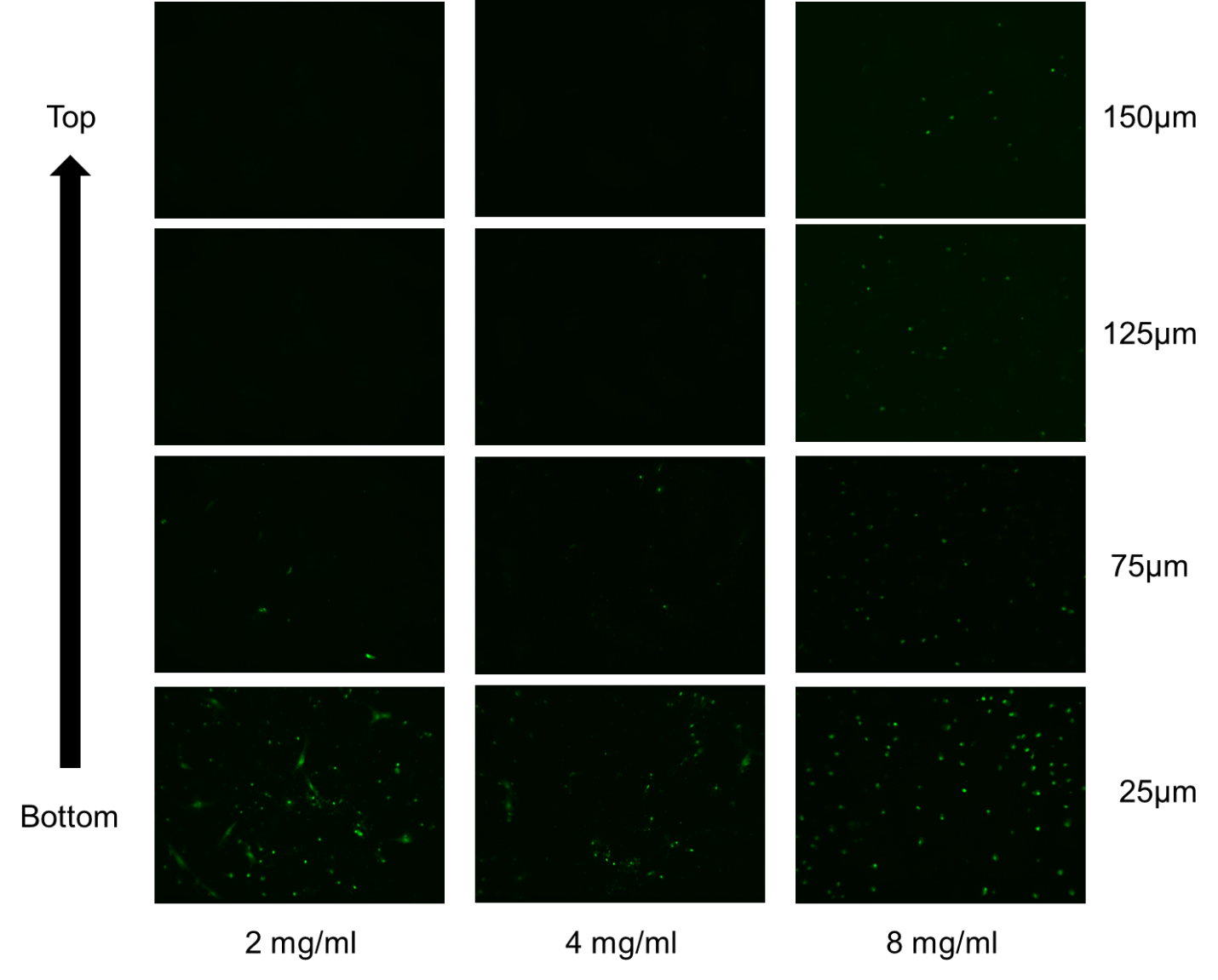


**Supplemental Fig. 2.** Representative images of YoPro^TM^ stained hRPCs within collagen type I hydrogel.


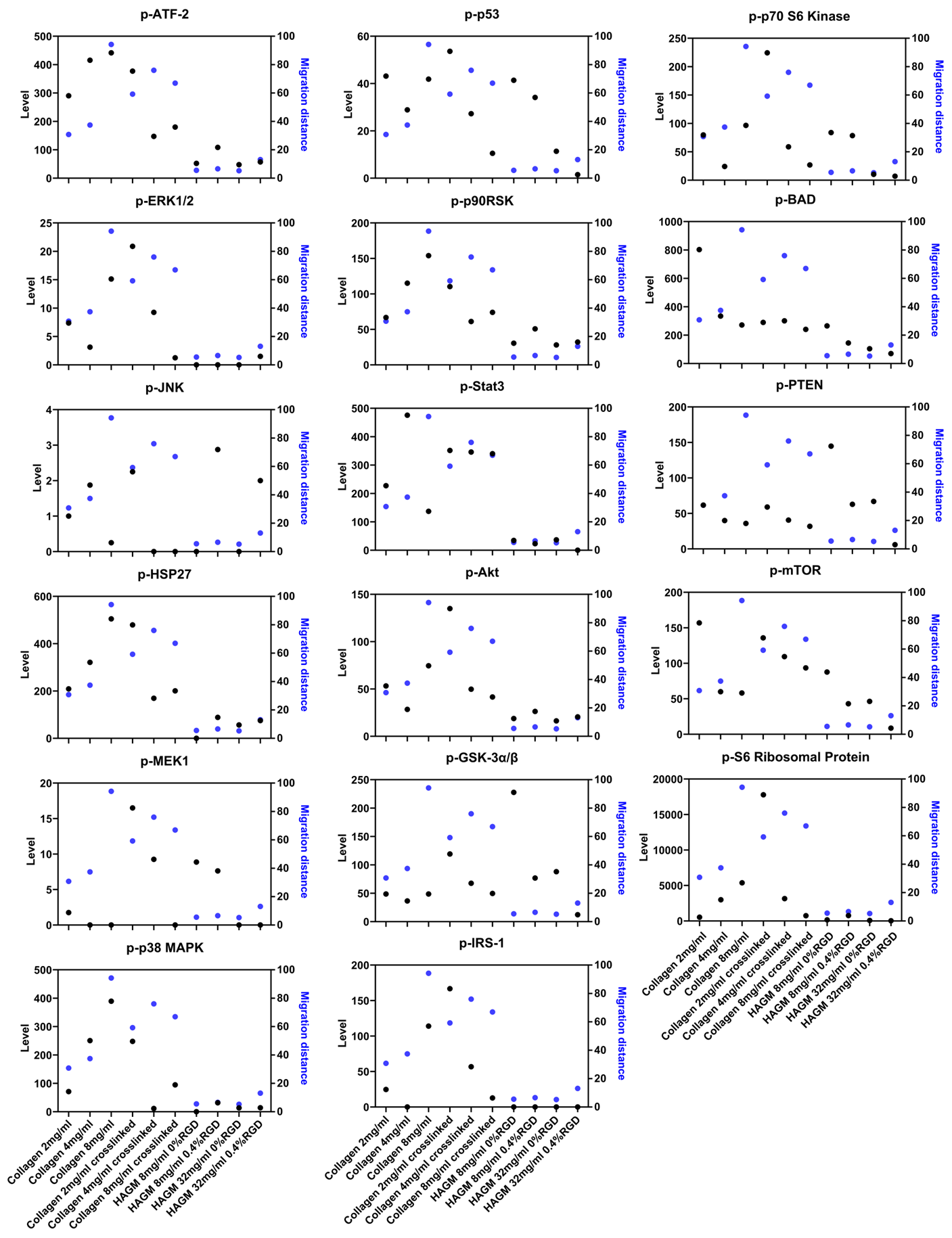


**Supplemental Fig. 3.** BioPlex data of 17 signaling nodes plotted with average migration distance in different hydrogels. Left Y axis represents phosphoprotein expression level (median fluorescence intensity, MFI) and right Y axis represents average migration distance (µm). From left to right on the X axis are collagen type I group (first 3 data points), collagen type I crosslinked group (second 3 data points), and HAGM group (last 4 data points).


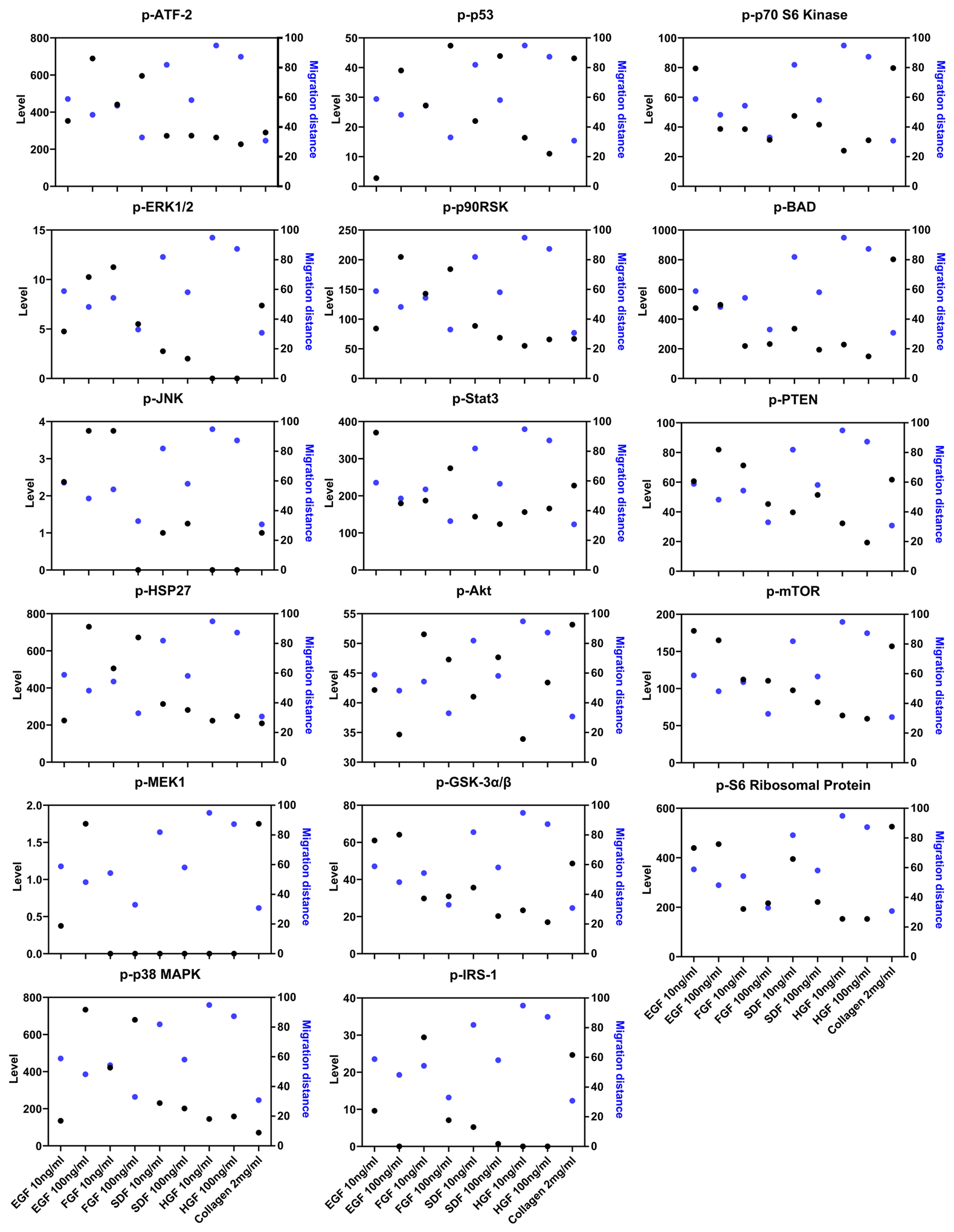


**Supplemental** **Fig. 4.** BioPlex data of 17 signaling nodes plotted with average migration distance in groups treated with different growth factors. Left Y axis represents phosphoprotein expression level (median fluorescence intensity, MFI) and right Y axis represents average migration distance (µm). From left to right on the X axis are EGF group (first 2 data points), FGF group (second 2 data points), SDF group (third 2 data points), HGF group (forth 2 data points), and 2 mg/ml collagen control group (last data point).


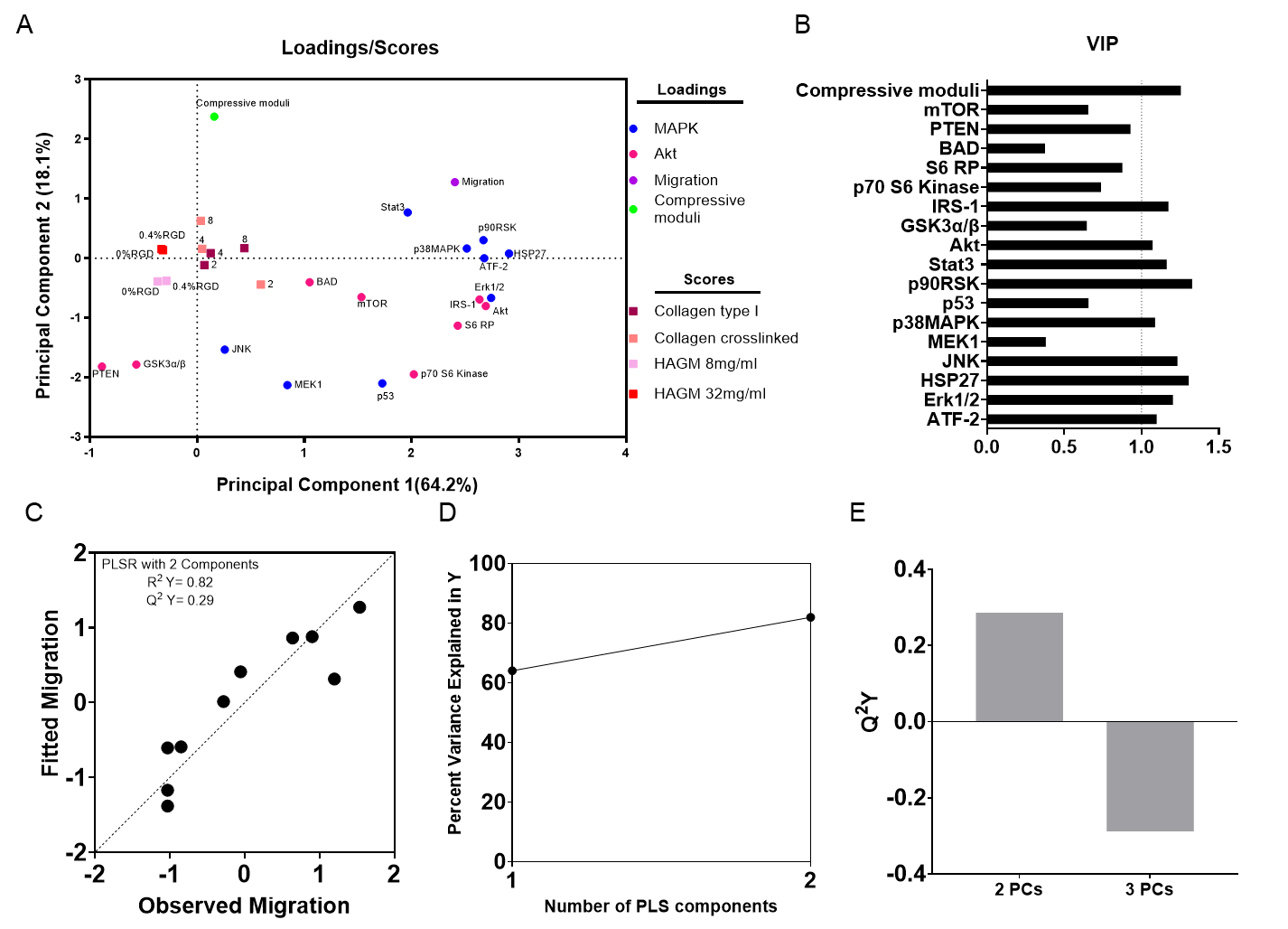


**Supplemental Fig. 5.** PLSR modeling with 2 principal components of impact of signaling nodes on migration distance in different hydrogel matrices. **(A)** Projection of loadings and scores onto the first two principal components. Loadings of individual phosphoprotein nodes were plotted in blue (MAPK) and red (Akt) circles. Loading of cell migration was plotted as a purple circle. Loading of compressive moduli was plotted as a green circle. Scores of each hydrogel formulation were plotted in squares. **(B)** VIP scores of 17 signaling nodes and compressive moduli. **(C)** Predicted migration vs. observed migration. Model quality and cross-validation prediction accuracy were determined by R^2^Y (0.82) and Q^2^Y (0.29). **(D)** Percent variance explained in Y. **(E)** Q^2^Y for each principal component.
