# Supplementary figures and images for: Human Retinal Progenitor Cell (hRPC) Migration in Three-Dimensional (3D) Environments of Varying Stiffness and Composition
Source: J Tissue Eng Regen Med. 2025 Oct 28;2025:9963972. doi: 10.1155/term/9963972 (PMC12585844; doi:10.1155/term/9963972)

## Slide 1
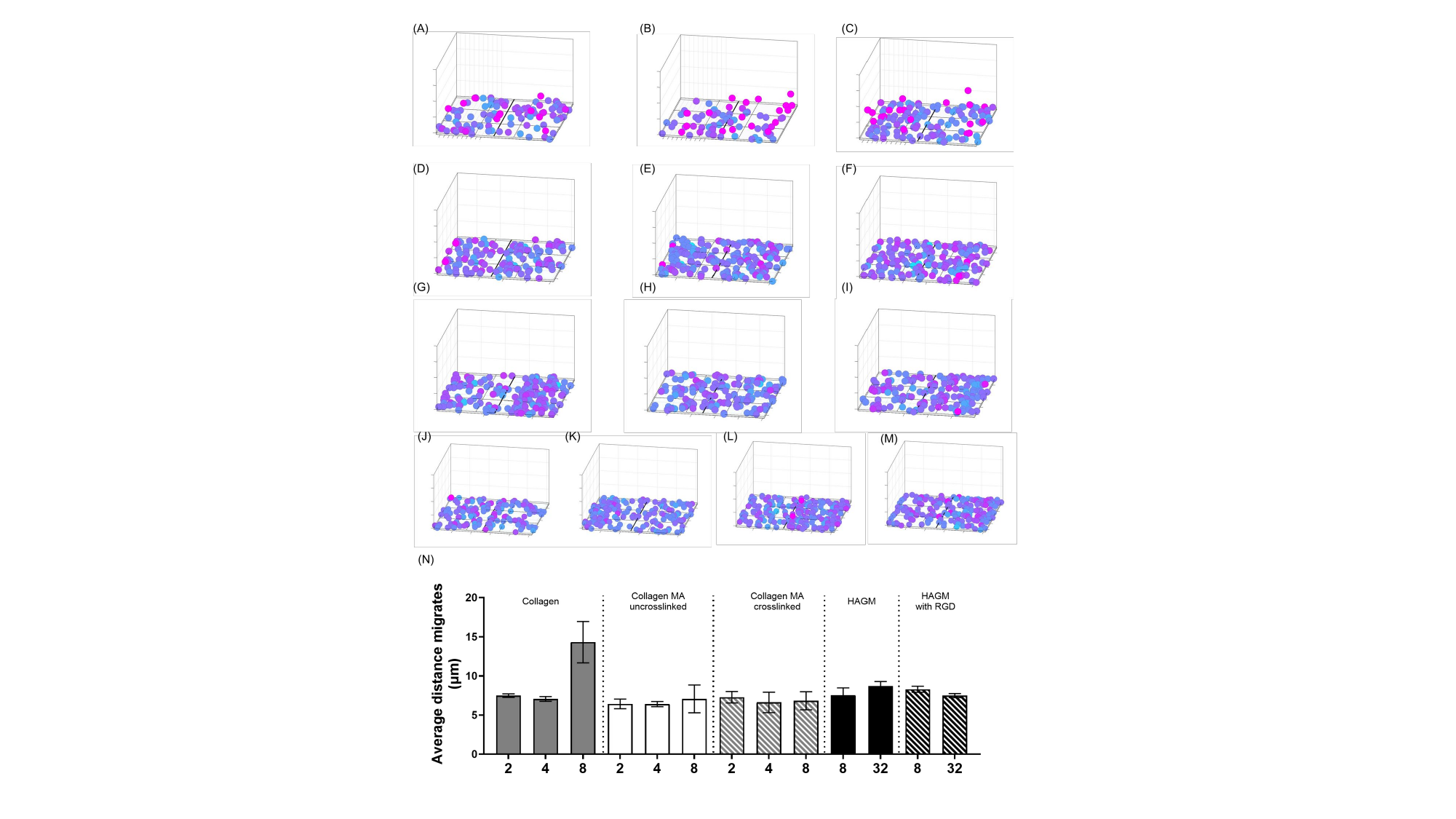

Supplement: Supporting Information — Additional supporting information can be found online in the Supporting Information section. [file 9963972.f1.zip › Supplemental Fig. 1.pptx]

## Slide 1
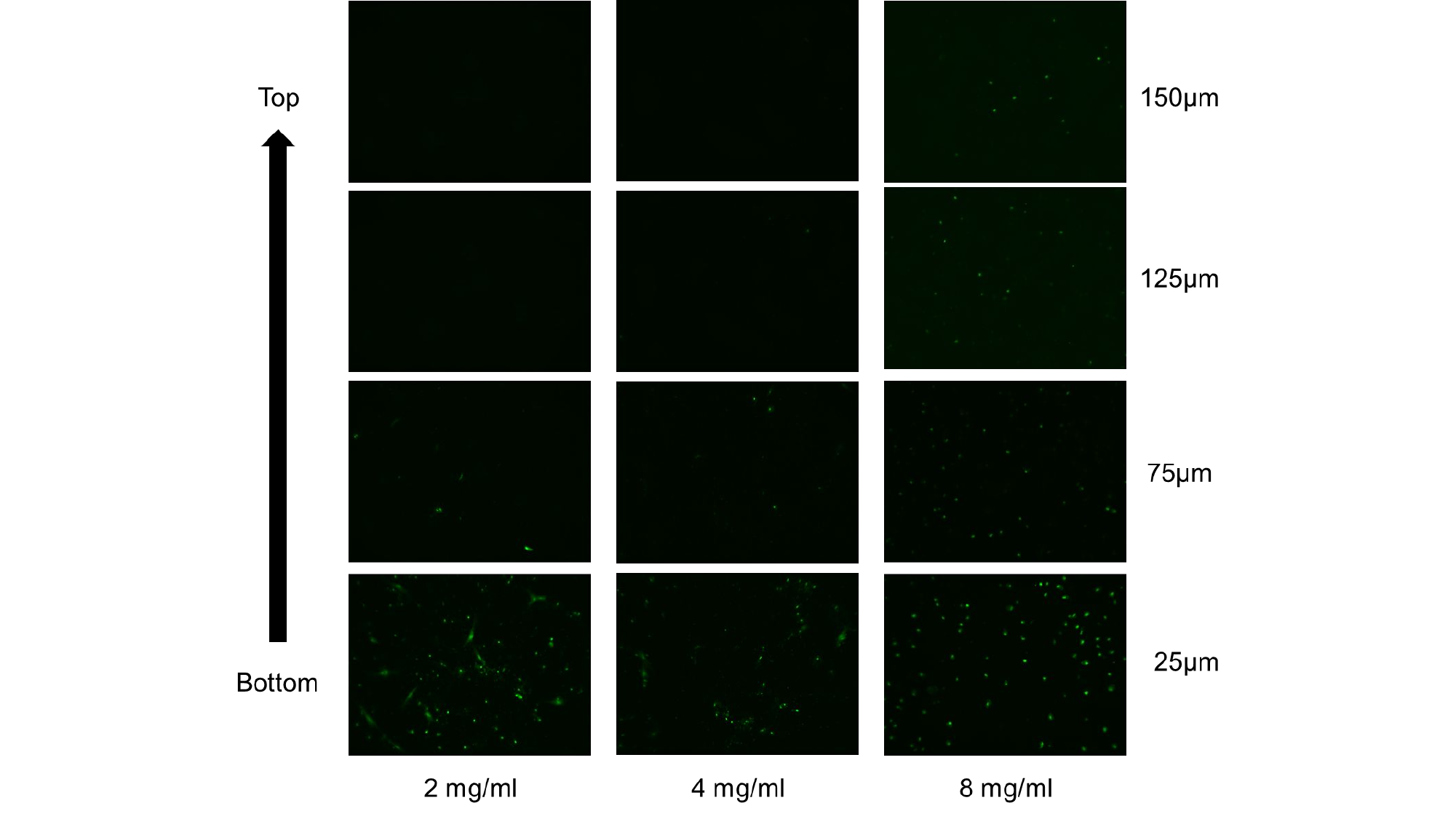

Supplement: Supporting Information — Additional supporting information can be found online in the Supporting Information section. [file 9963972.f1.zip › Supplemental Fig. 2.pptx]

## Slide 1
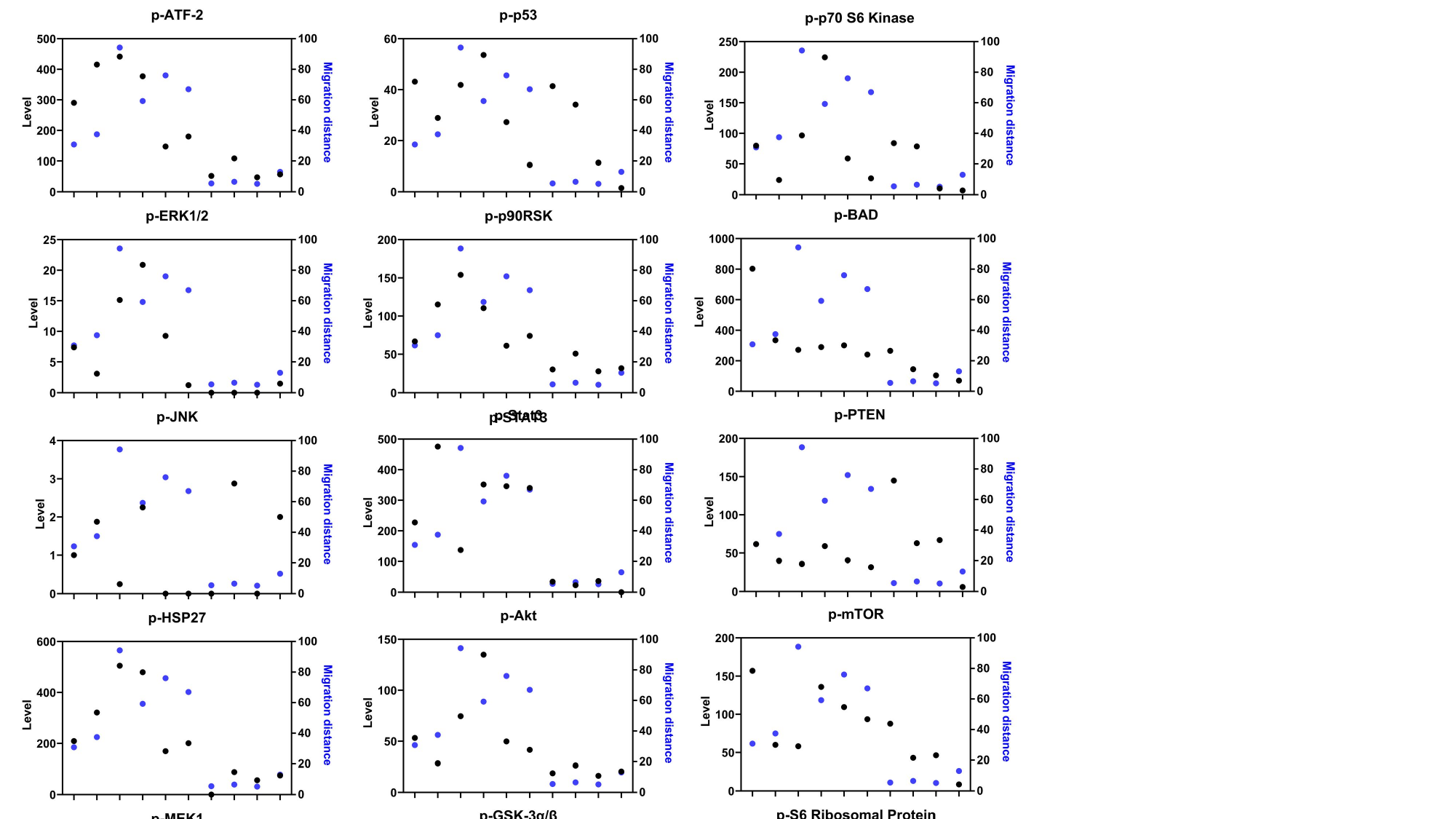

Supplement: Supporting Information — Additional supporting information can be found online in the Supporting Information section. [file 9963972.f1.zip › Supplemental Fig. 3_Revised.pptx]

## Slide 1
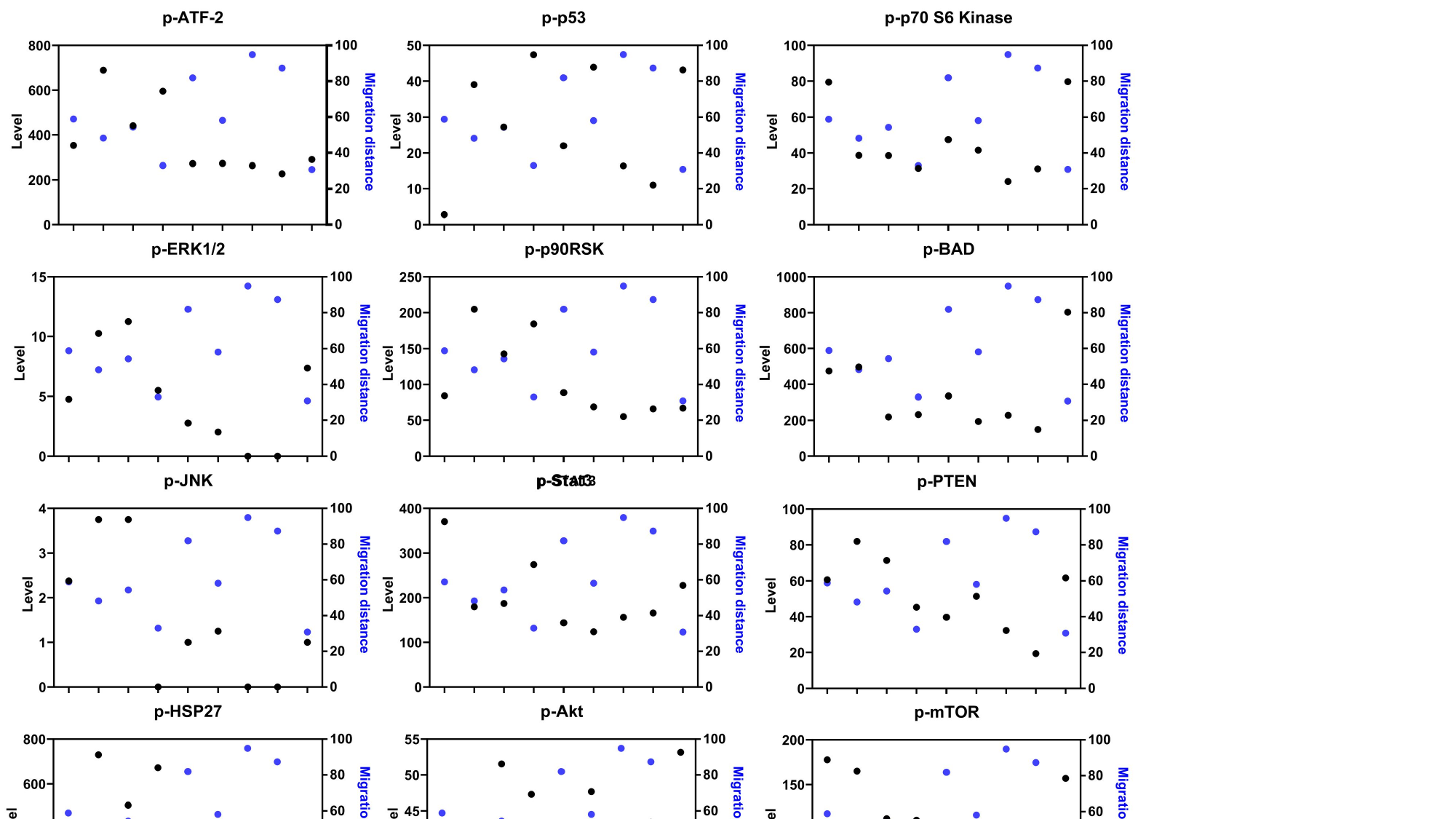

Supplement: Supporting Information — Additional supporting information can be found online in the Supporting Information section. [file 9963972.f1.zip › Supplemental Fig. 4_Revised.pptx]

## Slide 1
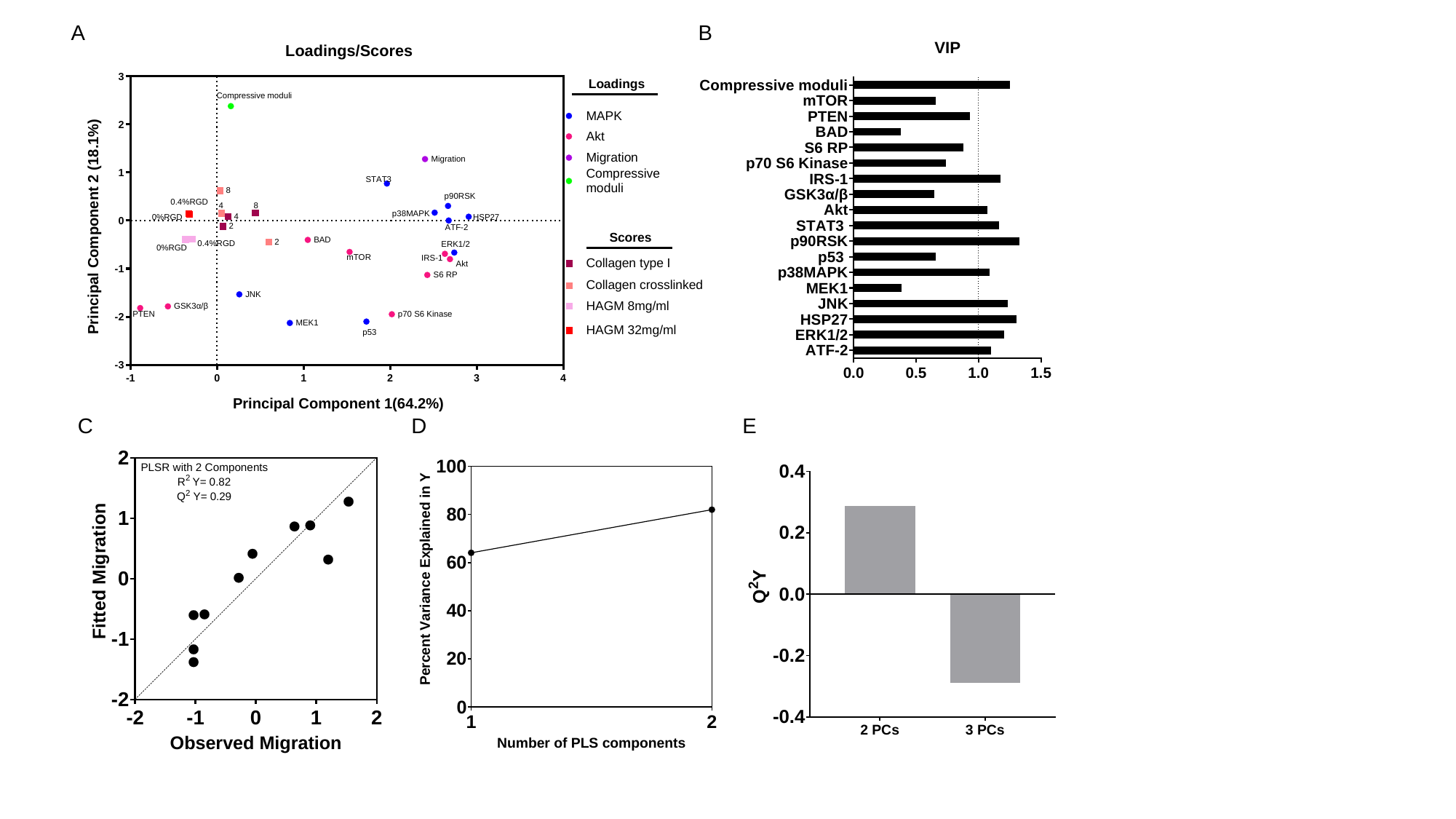

A
B
C
D
E

Supplement: Supporting Information — Additional supporting information can be found online in the Supporting Information section. [file 9963972.f1.zip › Supplemental Fig. 5_Revised.pptx]
